# Damage-associated molecular patterns and sensing receptors based molecular subtypes in malignant pleural mesothelioma and implications for immunotherapy

**DOI:** 10.3389/fimmu.2023.1104560

**Published:** 2023-03-24

**Authors:** Zheng Liu, Rui Wan, Hua Bai, Jie Wang

**Affiliations:** State Key Laboratory of Molecular Oncology, Department of Medical Oncology, National Cancer Center/National Clinical Research Center for Cancer/Cancer Hospital, Chinese Academy of Medical Sciences and Peking Union Medical College, Beijing, China

**Keywords:** malignant mesothelioma, damage-associated molecular patterns, immunogenic cell death, immunotherapy, tumor microenvironment

## Abstract

**Objectives:**

Malignant pleural mesothelioma (MPM) is characterized as an incredibly aggressive form of cancer with a dismal diagnosis and a dearth of specific biomarkers and therapeutic options. For MPM patients, the effectiveness of immunotherapy may be influenced by damage-associated molecular pattern (DAMP)-induced immunogenic cell death (ICD).The objective of this work is to create a molecular profile associated with DAMPs to categorize MPM patients and predict their prognosis and response to immunotherapy.

**Methods:**

The RNA-seq of 397 patients (263 patients with clinical data, 57.2% male, 73.0% over 60 yrs.) were gathered from eight public datasets as a training cohort to identify the DAMPs-associated subgroups of MPMs using K-means analysis. Three validation cohorts of patients or murine were established from TCGA and GEO databases. Comparisons were made across each subtype’s immune status, gene mutations, survival prognosis, and predicted response to therapy.

**Results:**

Based on the DAMPs gene expression, MPMs were categorized into two subtypes: the nuclear DAMPs subtype, which is classified by the upregulation of immune-suppressed pathways, and the inflammatory DAMPs subtype, which is distinguished by the enrichment of proinflammatory cytokine signaling. The inflammatory DAMPs subgroup had a better prognosis, while the nuclear DAMPs subgroup exhibited a worse outcome. In validation cohorts, the subtyping system was effectively verified. We further identified the genetic differences between the two DAMPs subtypes. It was projected that the inflammatory DAMPs subtype will respond to immunotherapy more favorably, suggesting that the developed clustering method may be implemented to predict the effectiveness of immunotherapy.

**Conclusion:**

We constructed a subtyping model based on ICD-associated DAMPs in MPM, which might serve as a signature to gauge the outcomes of immune checkpoint blockades. Our research may aid in the development of innovative immunomodulators as well as the advancement of precision immunotherapy for MPM.

## 1 Introduction

Mesothelioma is an unusual malignancy that originates from the mesothelial cells of the pleural or other regions. About 81% of the tumors originate from the pleura. The prevalence of malignant mesothelioma is increasing, but the mortality remains unchanged. In China, the incidence rate of malignant mesothelioma was only 1.50/10^6^ whereas the fatality rate was 1.22/10^6^ (1). MPM is mainly seen in older men exposed to asbestos. Compared with European and American countries, the onset age of mesothelioma is younger in China. The prevalence and fatality of malignant mesothelioma in China increase rapidly after the age of 35 or 40, reaching a peak at the age of 80 or 85 (1). Malignant pleural mesothelioma (MPM) is difficult to treat and has a dismal prognosis because most patients are at advanced stages when first diagnosed and are with early onset of evident clinical manifestations. Due to its resistance to conventional therapies and the absence of effective alternative regimens, MPM presents a highly difficult challenge. Despite the prompt advancement of immunotherapy and the fairly encouraging outcomes of ICIs in treating MPM, it remains high mortality on a global scale. The 5-year survival rate is around 10%, and the median overall survival is roughly one year.

Based on multiple studies conducted in MPM with immunotherapy alone or combined applied, the median PFS of 4~7 months does not seem particularly impressive. However, the increased median OS is mainly driven by a small portion of patients with long-lasting responses and deserves more explorations (2). CheckMate-743, a phase 3 randomized controlled trial, recently showed that MPM could benefit from PD-1 inhibitors combined with CTLA-4 inhibitors (3). Subgroup analyses revealed that the response rate to ICIs in MPM is somewhat but not entirely related to histology. Coupled with the fact that ICIs are more expensive and not covered by health insurance, it will result in a low cost-benefit ratio if the treatment is not effective. Therefore, there is an urgent need for identifying the subtypes of MPM patients who would potentially benefit from immunotherapy (4).

Immunogenic cell death (ICD) is a form of regulated cell death (RCD), acting as a major initiator of adaptive immune response in the context of malignant neoplasms (5). The promotion of ICD sensitizes MPM to ICIs treatments, as demonstrated by *in-vitro* experiments, preclinical models, and preliminary trials (6–11). which raises the possibility that ICD-associated biomarkers could serve as prospective predictive indicators for immunotherapy. An increased amount of work has discovered that induction of adaptive immune responses by cancer cells undergoing ICD is dependent on the emission and detection of a particular panel of DAMPs, including cell surface-exposed calreticulin (CALR), high mobility group box 1 (HMGB1) and extracellular adenosine triphosphate (ATP) (12, 13). In addition, previous studies also have demonstrated that ICD-associated DAMPs produced by chemotherapy or radiotherapy activate the cytotoxic CD8+ T cell and alleviate the immunosuppressive tumor microenvironment (TME), thus suggesting an essential role of DAMPs in immunotherapy (10). Pattern recognition receptors (PRRs) bound by DAMPs present adjuvanticity by activating transcription factors, eliciting APC cell activation, differentiation, and maturation, promoting the release of type 1 interferons and chemokines, resulting in the recruitment of APCs and T cells, and ultimately modulating intrinsic and adaptive immunity (14). Whether there is a pre-existing anti-tumor immune response is essential for effective immune checkpoint blockade. Effector T cells release interferon-γ (IFN-γ) by recognizing tumor neoantigens, which activates the Janus kinase (JAK)– signal transducer and activator of transcription (STAT) signaling pathway. The expression of programmed cell death ligand 1 (PD-L1) on the surface of tumor cells is mediated by the subsequent stimulation of the transcription factor interferon regulatory factor 1 (IRF1), which negatively regulates the effector T cell response in turn (15, 16). Immune checkpoint inhibitors (ICIs) disrupt this negative feedback loop as one of the primary mechanisms to restore anti-tumor immunity and exert anti-tumor efficiency. Hence, sensitizing tumors to ICIs by maneuvering ICD-associated DAMPs hinges on the inflammatory tumor immune microenvironment of MPM. Thus, we plan to assess the distinctive TiME to analyze the immune profiles of distinct MPM subtypes, which is crucial to interpret varied prognoses and efficacy of immunotherapy.

**Figure 1:**
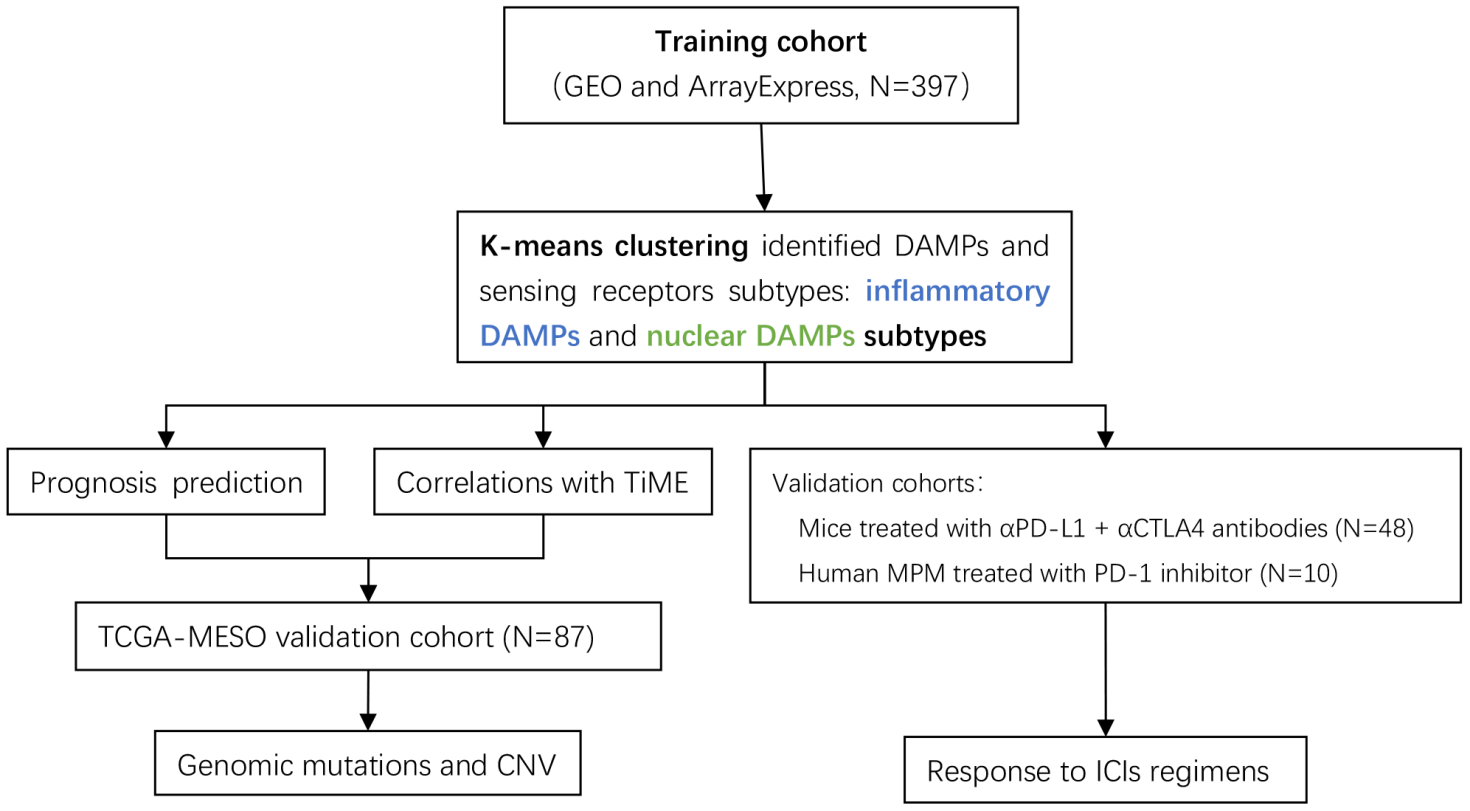
Flow chart of the data analysis process. The DAMPs-associated subtypes were established based on 397 TNBCs from the training cohort and validated in the TCGA cohort. DAMPs, damage-associated molecular patterns; TCGA, The Cancer Genome Atlas.

Despite the fact that an increasing amount of predictive models related to immunotherapy have been constructed to elaborate subtypes of MPM, ICD-associated DAMPs and their receptors were barely based upon to construct a predictive classification model. In this research, we performed consensus clustering analysis based on the ICD-associated DAMPs gene set and investigated the impact of DAMPs and their sensing receptors on the immune status from a variety of perspectives and on the survival expectancy of MPM patients. Additionally, the DAMPs-based classification we established was assessed for its predictive value of immune checkpoint inhibitors (ICIs) applied to mesothelioma (the flow chart of analysis is demonstrated in **Figure 1** **)**. Our research offers novel information to discover the potential molecular mechanisms in different subtypes of MPM, which may fulfill the demand for precision immunotherapy of MPM.

## 2 Materials and methods

### 2.1 Data collection

Normalized microarray gene expression data and clinical information of GSE42977, GSE2549, GSE12345, GSE51024, GSE163720, GSE163721, GSE29354, and GSE99070 were obtained from the GEO database (https://www.ncbi.nlm.nih.gov/geo/). Raw microarray gene expression data and follow-up data were downloaded from the ArrayExpress repository under accession code E-MTAB-6877 (https://www.ebi.ac.uk/biostudies/arrayexpress). TCGA sequencing data (including mRNA and genomic data) and clinical data of MPM patients were collected from Genomic Data Commons Data Portal (https://portal.gdc.cancer.gov/). Raw Next-Generation Sequencing (NGS) data of GSE117358 and GSE153941 were obtained from GEO database (https://www.ncbi.nlm.nih.gov/geo/).

Our study established one training cohort from malignant pleural mesothelioma patients from GEO and ArrayExpress datasets, including GSE42977, GSE2549, GSE12345, GSE51024, GSE163720, GSE163721, GSE29354 and E-MTAB-6877 (a total of 397 MPM patients, 57.2% male, 63 patients with overall survival data) and three validation cohorts consist of the TCGA-MESO datasets (a total of 86 MPM patients with CNV and WES data, 82.5% male, 86 patients with overall survival data), two murine GEO datasets (including GSE117358 and GSE153941), and one GEO dataset(GSE99070), respectively.

The raw data were normalized by using the RMA algorithm provided by “limma” package of R software (http://bioconductor.org/packages/limma/). Furthermore, the batch effect across datasets was subtracted using the “removeBatchEffect” function implemented in the “limma” package.

The demographic information and clinical characteristics of the training cohort are displayed in **Table 1**.

**Table 1:** Correlation between clinical characteristics and pathological features and DAMPs associated subtypes.

Features	Number of patients	DAMPs associated subtypes	
		Inflammatory DAMPs	Nuclear DAMPs
Total	397	230	167
Age			*P*=0.193
>60 years	46	27	19
≤60 years	17	13	4
Gender			*P*=0.719
Male	111	71	40
Female	83	51	32
Stage			*P*=0.982
I	2	2	0
II	7	4	3
III	25	14	11
IV	17	11	6
Histology			*P*>0.05
Epithelial	88	51	37
Biphasic	17	10	7
Sarcomatoid	10	6	4
DMM	1	0	1
Asbestos exposure			*P*>0.05
Exposed	44	28	16
Not exposed	13	7	6
Probably exposed	3	2	1

### 2.2 Identification of DAMPs subgroups by K-means analysis

32 DAMPs-related genes were collected according to previous research (5, 17, 18). 25 DAMPs-related genes were contained in the training and validation cohorts and their information is shown in **Table 2**.

**Table 2:** DAMPs-associated genes.

Gene	Protein	Molecular type	Function (s)
*TLR4*	Toll-like receptor 4	PRRs (TLRs)	Tumor Antigen processing and presentation
*TLR2*	Toll-like receptor 2	PRRs (TLRs)	NLRP3 inflammasome activation
*CLEC4E*	C-type lectin domain family 4 member E	PRRs (CLRs)	Activates innate immune receptors on monocytes, macrophages, and immature dendritic cells
*CLEC7A*	C-type lectin domain family 7 member A(Dectin-1)	PRRs (CLRs)	Recognize a variety of glucans to activate innate immune response
*NLRP3*	NOD-like receptor thermal protein domain associated protein 3 (cryopyrin)	PRRs (NLRs)	Regulates inflammation, the immune response, and apoptosis
*FPR1*	Formyl peptide receptor 1	PRRs (GPCRs)	Guide phagocytic leukocytes to regions of inflammation (19)
*AIM2*	Absent in melanoma 2	PRRs (ALRs)	Initiates inflammasome assembly in response to DNA damage (20, 21)
*IFIH1*	Interferon induced with helicase c domain 1	PRRs (RLRs)	Promotes the production of IFN-I and cytokines
*DDX58*	Retinoic acid-inducible gene I protein	PRRs (RLRs)	Trigger a transduction cascade
*FPR2*	Formyl peptide receptor 2	PRRs (GPCRs)	Regulates monocyte chemotaxis
*TLR7*	Toll-like receptor 7	PRRs (TLRs)	Stimulates autoreactive B cells (22, 23)
*TLR3*	Toll-like receptor 3	PRRs (TLRs)	Promotes type I IFN secretion; initiates CXCL10 release (24–26)
*IL33*	Interleukin 33	DAMPs	Involves the activation of natural killer cells (27–29)
*TREM1*	Triggering receptor expressed on myeloid cells 1	PRRs (TREMs)	Triggers pro-inflammatory cytokine and chemokine secretion; enhanced inflammatory responses (30–32)
*BCL2*	Apoptosis regulator Bcl-2	DAMPs	Blocks the apoptotic death of lymphocytes (33)
*CASR*	Extracellular calcium-sensing receptor	PRRs (GPCRs)	Promotes NLRP3 activation
*AGER*	Advanced glycosylation end product-specific receptor	PRRs	Elevates pro-inflammatory genes expression
*IL1A*	Interleukin-1 alpha	DAMPs	Cell activation, cytokine release
*CALR*	Calreticulin	DAMPs	Promotes the uptake of dying cells and type I IFN secretion by APCs (34–36)
*ROCK1*	Rho-associated protein kinase 1	DAMPs	Regulates focal adhesions of fibroblasts and gathering of lymphocytes (37)
*HSP90AA1*	Heat shock protein HSP 90-alpha	DAMPs	Assists the proper folding of specific proteins through ATPase activity (38, 39)
*PANX1*	Pannexin-1	DAMPs	Mediates ‘find-me’ signal release during apoptosis (40, 41)
*PPIA*	Peptidyl-prolyl cis-trans isomerase A	DAMPs	Assists to activate the tyrosine kinase Jak2 (42)
*HMGN1*	Non-histone chromosomal protein HMG-14	DAMPs	Promotes B cell proliferation (43–45)
*HSPA4*	Heat shock 70 kDa protein 4	DAMPs	Enable ATP binding activity (46)

R package “ConsensusClusterPlus” based on the DAMPs-related gene list expressed in cohorts were employed to conduct unsupervised clustering. K-means clustering (the “kmeans” algorithm in R) was performed to define stable DAMPs-associated subtypes of MPM.

### 2.3 Signaling pathways analyses

Differentially expressed genes (DEGs) between two groups were defined as genes whose false discovery rate (FDR) value was < 0.05 and |Log2 (Fold Change (FC))|> 1.

Metascape database was used for Gene Ontology (GO) and Kyoto Encyclopedia of Genes and Genomes (KEGG) enrichment analysis (47).

Furthermore, gene set enrichment analysis (GSEA) and gene set variation analysis (GSVA) were employed in two subtypes to exploit the differences in mechanisms (48–50). An adjusted P-value < 0.05 was deemed as statistically significant.

The gene sets utilized for GSEA and GSVA were downloaded from the MSigDB database.

### 2.4 Immune status analyses

CIBERSORT was applied to characterize immune infiltrating cell type proportions in expression profiles using a validated leukocyte gene signature matrix (LM22) (51).

The R package “estimate” contains the Estimation of Stromal and Immune cells in Malignant Tumor tissues using Expression data (ESTIMATE) program that derived the immune score (52).

### 2.5 Genomic analyses

Copy number variations (CNV) and genomic mutations were analyzed using GISTIC2.0 in TCGA-MESO cohort (53). We depicted the variances in gene amplification or deletion events and genomic mutations between DAMPs-associated MPM subtypes.

The “ComplexHeatmap” package in R was implemented to visualize the waterfall plot of CNV and genomic mutation data (54).

### 2.6 Statistical analyses

Statistical analyses were conducted by R (version 4.1.1, https://www.r-project.org).

The Kaplan–Meier algorithm included in the “survival” R package was used to perform the survival analysis.

For the comparison of the two groups, one-way analysis of variance (ANOVA), Chi-square test, or Fisher exact test was performed.

Mantel-Haenszel test was used to analyze the rates of occurrence of death over time.

A *P*<0.05 was deemed statistically significant.

## 3 Results

### 3.1 Consensus clustering identified two DAMPs-associated subtypes

**Figure 2:**
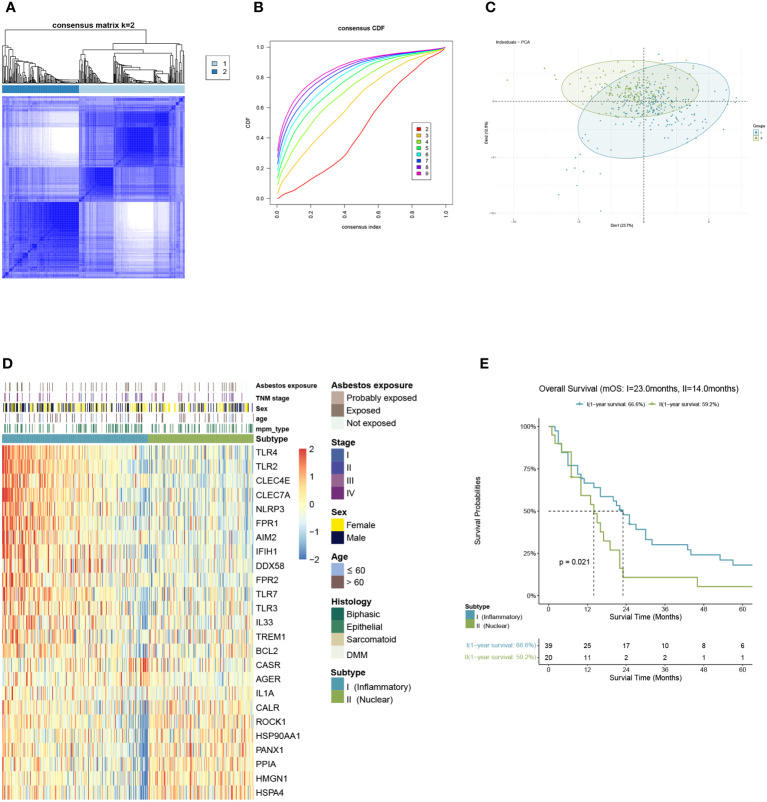
Identification of DAMPs-associated subtypes by K-means analysis. **(A–C)** K=2 was identified as the optimum value for consensus clustering. **(D)** DAMPs-associated subtyping of MPM patients (n = 397) from the training cohort. Heatmap displays normalized enrichment scores of the two subtypes. **(E)** Kaplan-Meier curves of overall survival (OS) between the two subtypes in the training cohort.

We included malignant pleural mesothelioma samples (n=397) from GEO and ArrayExpress datasets as the training cohort. Based on the gene expression related to DAMPs, they were divided into two subtypes *via* K-means clustering selecting 2 as the ideal and meaningful value of K (**Figures 2A–C**).

Of the 397 MPM patients included in the training cohort, 167 patients were classified into the nuclear DAMPs subgroup, and 230 were clustered in the inflammatory DAMPs subgroup. The heatmap reveals significant differences between the two subtypes in normalized enrichment scores of genes associated with DAMPs (**Figure 2D**). Cluster I is classified as the inflammatory DAMPs subtype distinguished by increased expression of PRRs regulating activities of inflammasome or immune cells, such as *TLRs, FPR1, CLEC4E, NLRP3*, etc. Cluster II is defined as the nuclear DAMPs subtype, with nuclear-associated DAMPs, such as *HSP90AA1, HSPA4, CALR* and *high-mobility group nucleosome binding protein 1 (HMGN1)* generally overexpressed, but the receptors being expressed at low levels. According to Kaplan–Meier survival analysis revealed that MPM patients enjoyed a better overall survival (OS) in the inflammatory DAMPs subtype, whereas patients in the nuclear DAMPs subtype had a worse prognosis (median overall survival 23.0months vs. 14.0months, *P*=0.021; **Figure 2E** **)**.

### 3.2 Identification of differentially expressed genes and enrichment of signal pathways in different DAMPs-associated subtypes

We detected DEGs between tumor tissues belonging to two subtypes and normal pleural tissues respectively and then conducted GSEA analysis to investigate their putative signaling pathways. A total of 327 DEGs were identified, among which 311 were belonged to the nuclear DAMPs subtype, and 219 were resided in the inflammatory DAMPs subtype.

**Figure 3:**
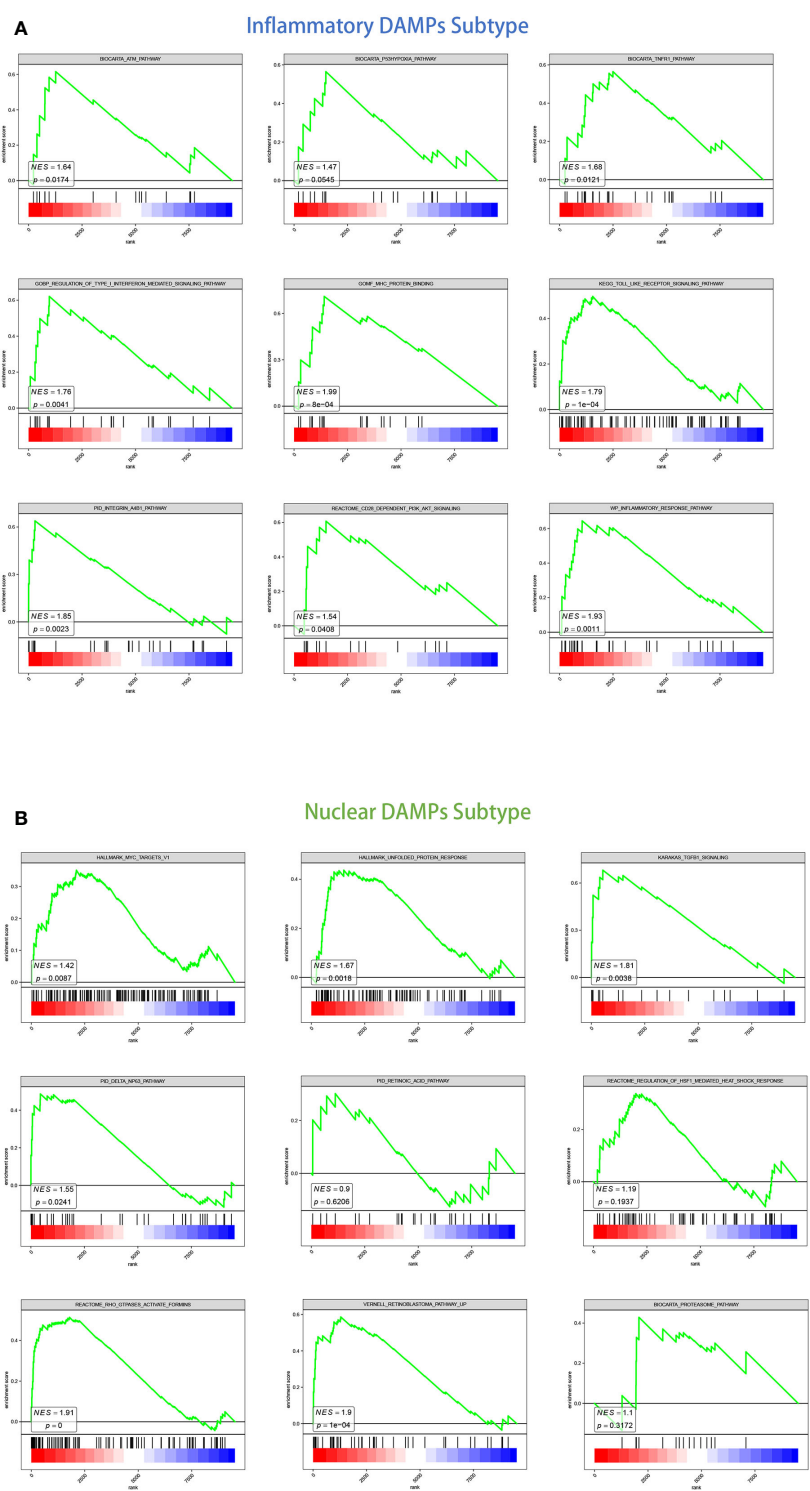
GSEA analysis of DEGs in two subtypes. **(A)** GSEA analysis of canonical pathways, gene ontology, hallmark gene sets for patients in the inflammatory DAMPs subtype. **(B)** GSEA analysis of canonical pathways, gene ontology, hallmark gene sets for patients in the nuclear DAMPs subtype.

For the inflammatory DAMPs subtype, pro-inflammatory pathways were primarily enriched. As shown by KEGG enrichment analysis, the DEGs were intensely enriched in the toll-like receptor (TLR) signaling pathway. The DEGs were observed to be enriched in immune-related signaling pathways by GO enrichment analysis, including MHC protein binding, tumor necrosis factor receptor (TNFR) binding, and regulation of type I interferon-mediated signaling. GSEA similarly revealed that the subtype of inflammatory DAMPs exhibited strong upregulation of the ATM pathway, TNFR1 pathway, FGF pathway, inflammatory response pathway, CD28-dependent PI3K-AKT signaling pathway, and integrin-A4B1 pathway **(** **Figure 3A** **)**.

Based on GSEA enrichment analysis, on the other hand, the DEGs were comparatively elevated in heat shock protein (HSP)-related signaling and immune-suppressed pathways for the nuclear DAMPs subtype, including proteasome pathway, unfolded protein response (UPR), TGFB1 signaling, Rho GTPases activate formins and regulation of HSF1-mediated heat shock response. The retinoblastoma pathway, delta NP63 pathway, MYC targets, defective intrinsic pathway for apoptosis, and retinoic acid pathway were also enriched significantly indicating that the nuclear DAMPs subtype proliferates fiercely (**Figure 3B**).

**Figure 4:**
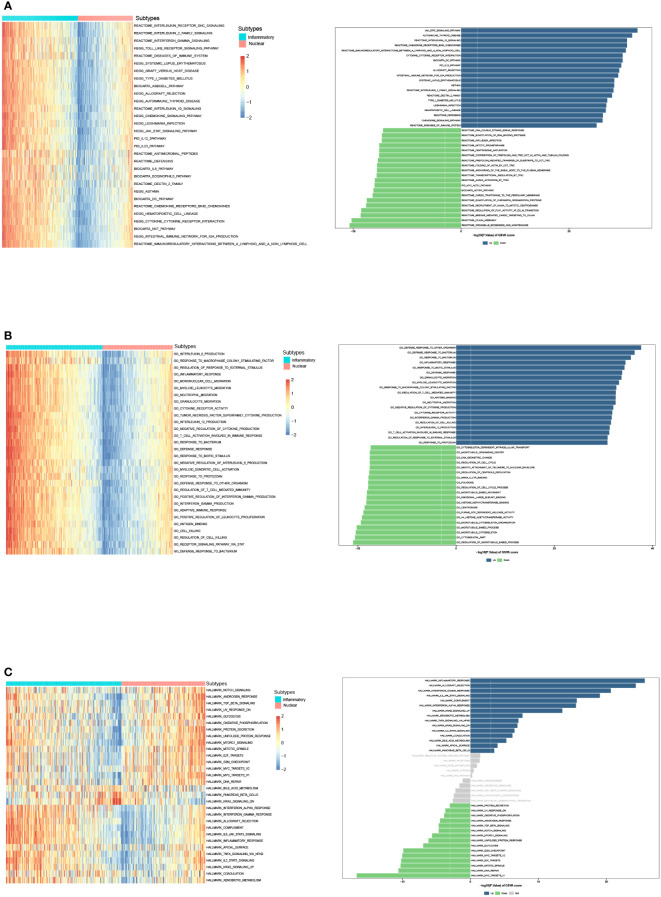
GSVA analysis of DEGs in the inflammatory DAMPs and nuclear DAMPs subtype. **(A)** GSVA analysis of canonical pathways (BIOCARTA, KEGG, PID, REACTOME and WIKIPATHWAYS) for patients in the inflammatory DAMPs and nuclear DAMPs subtype. **(B)** GSVA analysis of gene ontology (GO) for patients in the inflammatory DAMPs and nuclear DAMPs subtype. **(C)** GSVA analysis of hallmark gene sets for patients in the inflammatory DAMPs and nuclear DAMPs subtype.

We then identified DEGs between two subtypes, and GSVA was performed to compare the significantly differential pathway (**Figures 4A–C**). GSVA analysis of cancer hallmarks, canonical pathways, and gene ontology revealed that natural killer T cell (NKT), DC, and T cell activation, complement, inflammatory response, adaptive immune response, antigen binding, cytokine receptor, IL-2 family, IL-12, IFN-γ, TNF superfamily, TLR, chemokine signaling, IL6-JAK-STAT signaling pathways were triggered in the inflammatory DAMPs subtype. In contrast, the DAMPs in the nuclear DAMPs subtype triggered cancer hallmark MYC targets, NOTCH signaling, TGF-β signaling, unfolded protein response (UPR), MTORC1 signaling, and IL-8 production.

### 3.3 Immune statuses of the patients in the two molecular subtypes varied

**Figure 5:**
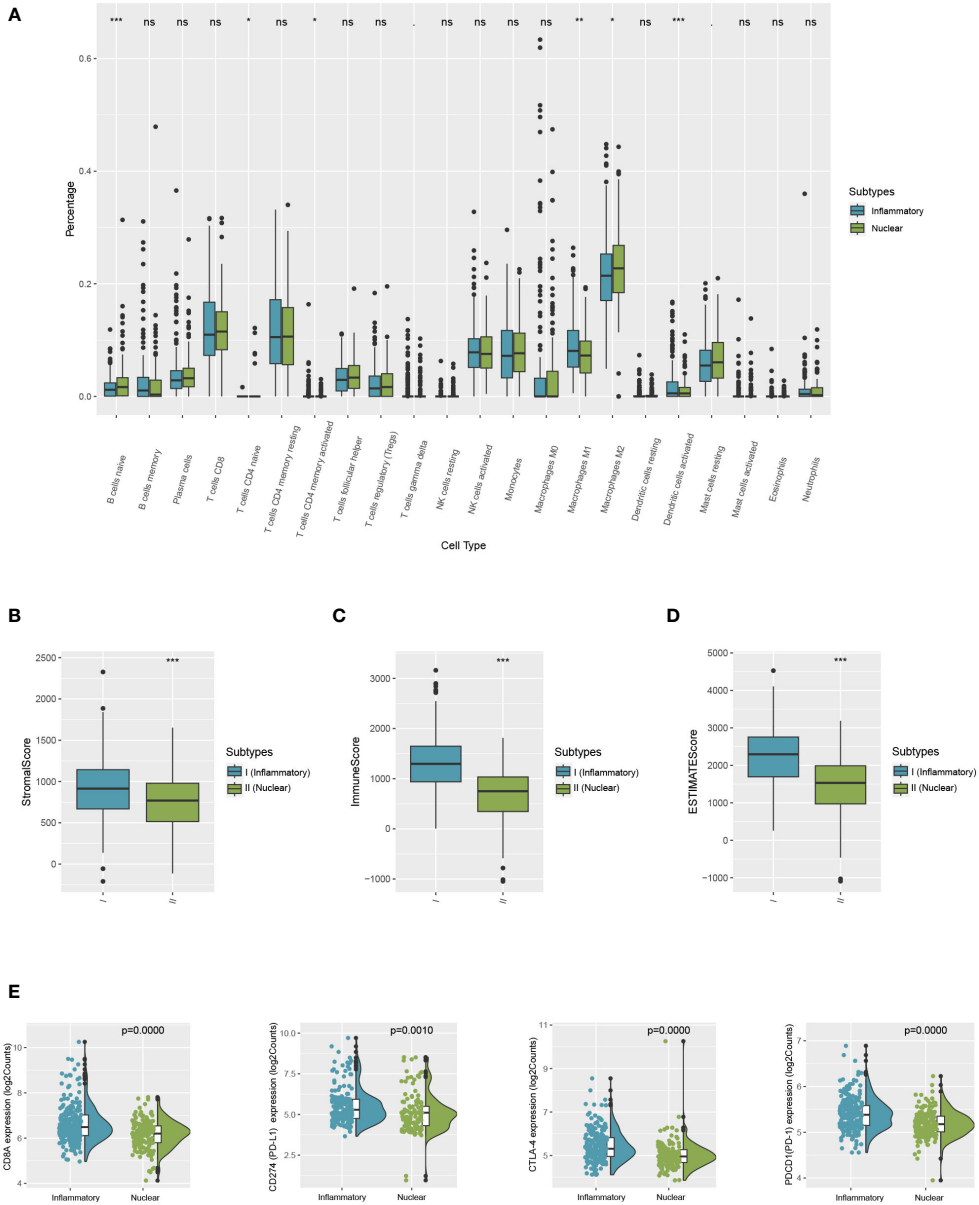
Comparison of differences between two subtypes in immune microenvironment status and immune signature expression levels. **(A)** The differential estimated proportion of 22 CIBERSORT immune cell types in DAMPs-associated subtypes. The central line represents the median value. The bottom and top of the boxes are the 25th and 75th percentiles (interquartile range). **(B)** Stromal score in DAMPs-associated subtypes. **(C)** Immune score in DAMPs-associated subtypes. **(D)** ESTIMATE score difference between the two subtypes in the training cohort. **(E)** Expression differences in CD8A, PD-1, PD-L1 and CTLA4 between two subtypes. *P < 0.05; **P < 0.01; ***P < 0.001; ns, not significant.

The immune variances between the two subtypes were investigated by immune analysis. The infiltration ratio of 22 immune cell types was analyzed in the training cohort using CIBERSORT and compared between the two groups. As demonstrated in **Figure 5**, the predominant infiltrating immune cells in the inflammatory DAMPs subtype were memory CD4+ T cells, while M1-like macrophages and activated DCs had a tendency to infiltration more, whereas Treg cells increased significantly with M2-like macrophages showed a tendency of higher infiltration in the nuclear DAMPs subtype (**Figure 5A**). Moreover, ESTIMATE indicated that patients within the inflammatory DAMPs subtype had considerably greater stromal, immune, and ESTIMATE scores compared to the others (**Figures 5B–D**). Additionally, the expression of CD8A (*P*=0.0000), PD-1 (*P*=0.0000), PD-L1 (*P*= 0.0010), and CTLA4 (*P*=0.0000) in the inflammatory DAMPs subtype were also notably greater than in the nuclear DAMPs subtype **(** **Figure 5E**), implying that PD-1/PD-L1 and CTLA4 may be the biomarkers of immune checkpoint inhibitors efficacy in MPM.

### 3.4 Solid validation of the immune characteristics in the TCGA-MESO cohort

**Figure 6:**
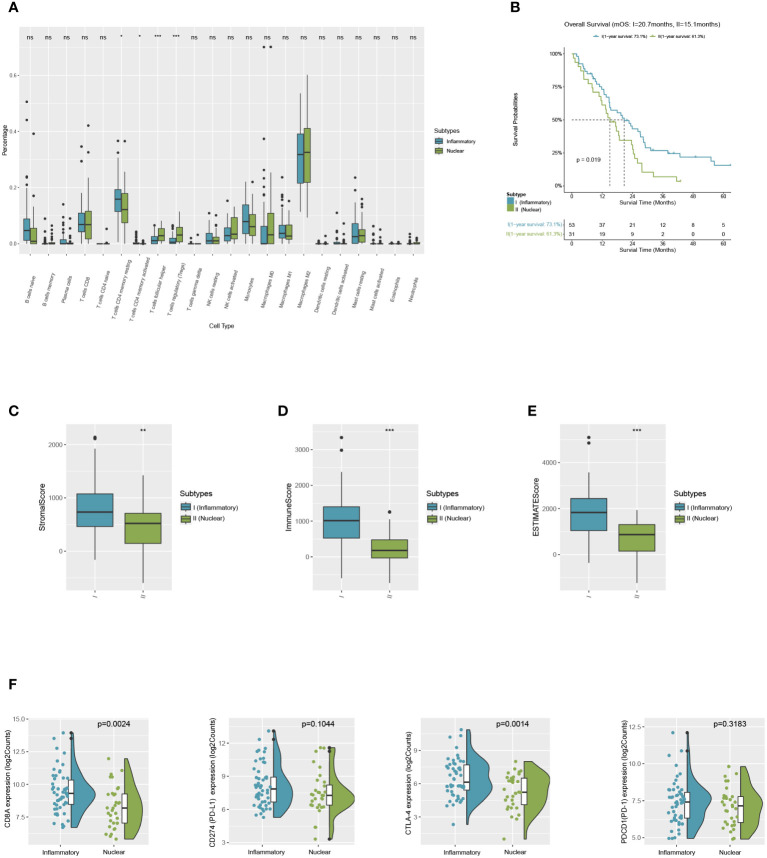
Successful validation of DAMPs-associated subtypes in the validation cohort of TCGA. **(A)** The differential estimated proportion of 22 CIBERSORT immune cell types in DAMPs-associated subtypes. The central line represents the median value. The bottom and top of the boxes are the 25th and 75th percentiles (interquartile range). **(B)** Kaplan-Meier curves of overall survival (OS) between the two subtypes in the validation cohort of TCGA. **(C)** Stromal score in DAMPs-associated subtypes. **(D)** Immune score in DAMPs-associated subtypes. **(E)** ESTIMATE score difference between the two subtypes in the validation cohort of TCGA. **(F)** Expression differences in CD8A, PD-1, PD-L1 and CTLA4 between two subtypes. *P < 0.05; **P < 0.01; ***P < 0.001; ns, not significant.

Patients of MPM were selected from the TCGA dataset as a validation cohort and they were segregated into two DAMPs-associated subgroups based on the processed algorithm in order to further test whether the features we outlined in the two subtypes of the training cohort could be generalized. By applying CIBERSORT as above, we inferred that M2-like macrophages in the nuclear DAMPs subtype infiltrated substantially and that activated memory CD4+ T cells, M1-like macrophages, and activated DCs infiltrated in the inflammatory DAMPs subtype increased significantly (**Figure 6A**). These findings are comparable to those of the training cohort. Additionally, patients of the inflammatory DAMPs subtype also exhibited better immune, stromal, and ESTIMATE scores (**Figures 6C–E** **)**.

In terms of survival analysis, patients of the inflammatory DAMPs subtype exhibited a superior OS than those in the nuclear DAMPs subtype (median overall survival 20.7months vs. 15.1months, *P*=0.019; **Figure 6B**). Additionally, the expression of CD8A (*P*=0.0024) and CTLA-4(*P*=0.0014) was notably higher in the inflammatory DAMPs subtype than in the nuclear DAMPs subtypes of TCGA cohort, while no statistical significance was found in terms of PDCD1 (PD-1) and CD274 (PD-L1) (**Figure 6F**), indicating that PD-L1 is possibly not the optimal indicators for ICIs effectiveness in MPM.

### 3.5 Contrast of genomic variations in two subgroups in the TCGA-MESO cohort

**Figure 7:**
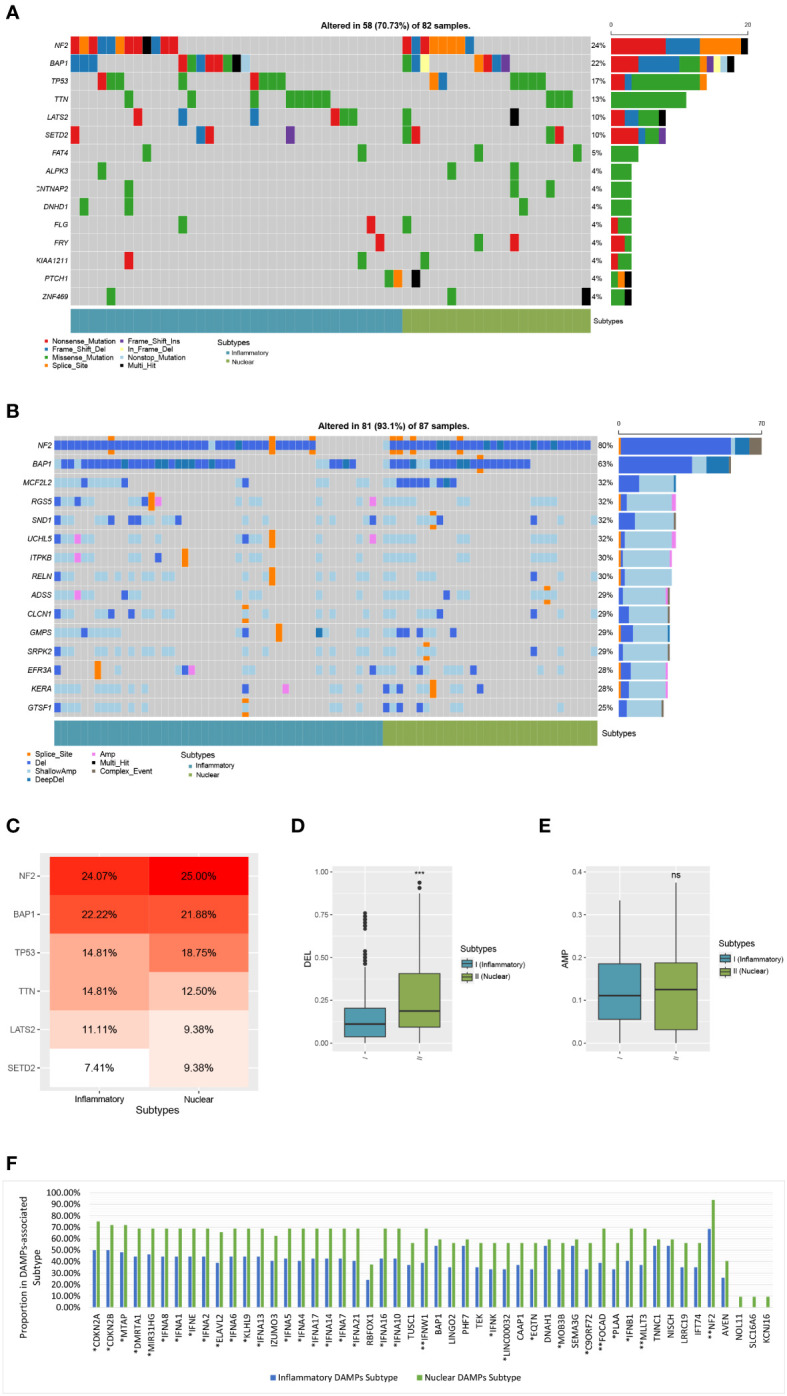
Comparison of genomic alterations of DAMPs-associated subtypes in the validation cohort of TCGA. **(A)** Differential somatic mutation analysis between the two subgroups. **(B)** Differential CNV analysis between the two subgroups. **(C)** Somatic mutation percentage of mostly mutated genes. **(D)** Arm-level copy number deletion in DAMPs-associated subtypes. **(E)** Arm-level copy number amplification in DAMPs-associated subtypes. **(F)** Comparison of Genes with copy number deletion in DAMPs-associated subtypes. *P < 0.05; **P < 0.01; ***P<0.001; ns, not significant.

We employed waterfall plots in order to detect genomic mutations and copy number variations between the two subgroups in the TCGA (**Figures 7A, B**). Although *TTN* mutations were more frequently detected in the inflammatory DAMPs subtype with genetic alterations (21.6% vs 14.3%, *P*=0.73; **Figure 7C**) and *TP53* mutations in nuclear DAMPs subtypes (21.6% vs 28.6%, *P*=0.55; **Figure 7C**), it did not achieve statistical significance, which may still be a hint to the significant prolonged OS of inflammatory DAMPs subtypes.

Moreover, the ratio of patients which have a copy number deletion in the nuclear DAMPs subtype was greater than in the inflammatory DAMPs subtype (*P*<0.001; **Figure 7D**) while the frequency of amplification between the two subtypes has no significant difference (*P*>0.05; **Figure 7E**). Deletions in genes were more frequently observed in the nuclear DAMPs subtype than in the inflammatory DAMPs subtype significantly, among which copy number loss in NF2 presented at the highest frequency (**Figure 7F**). Deletion was observed in MTAP, FOCAD, MLLT3 and type I IFN (e.g., IFNA1/2/4-10/13/14/16/17/21, IFNB1, IFNE, IFNK, IFNW1, and etc.) but at lower frequencies. Deletions on other genes were also analyzed as displayed in **Figure 7F**.

### 3.6 Prediction of immunotherapy efficacy in two subtypes

**Figure 8:**
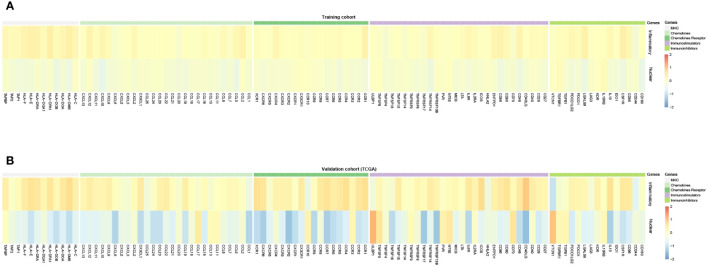
**(A)** Expression of MHC, chemokines and receptors, and immunomodulatory molecules for DAMPs-associated subtypes in the training cohort. **(B)** Expression of MHC, chemokines and receptors, and immunomodulatory molecules for DAMPs-associated subtypes in the TCGA cohort.

Furthermore, we evaluated the predictive value of immunotherapy efficacy between two subtypes. The expression of major histocompatibility complex (MHC), and cytokines and their receptors in two subtypes exhibited by heatmap (**Figures 8A, B**). It notably demonstrated that MHC molecules, immunostimulatory and immunoinhibitory molecules, and cytokines and their receptors are unevenly expressed in distinct subtypes, with the higher expressions, especially of CXCL10 and its receptor CXCR3, in the inflammatory DAMPs subtype, both in the training cohort and the validation cohort of TCGA. In the contrast, the expression of TGFB and TGFBR elevated in the nuclear DAMPs subtype, consistent with the GSEA and GSVA results.

**Figure 9:**
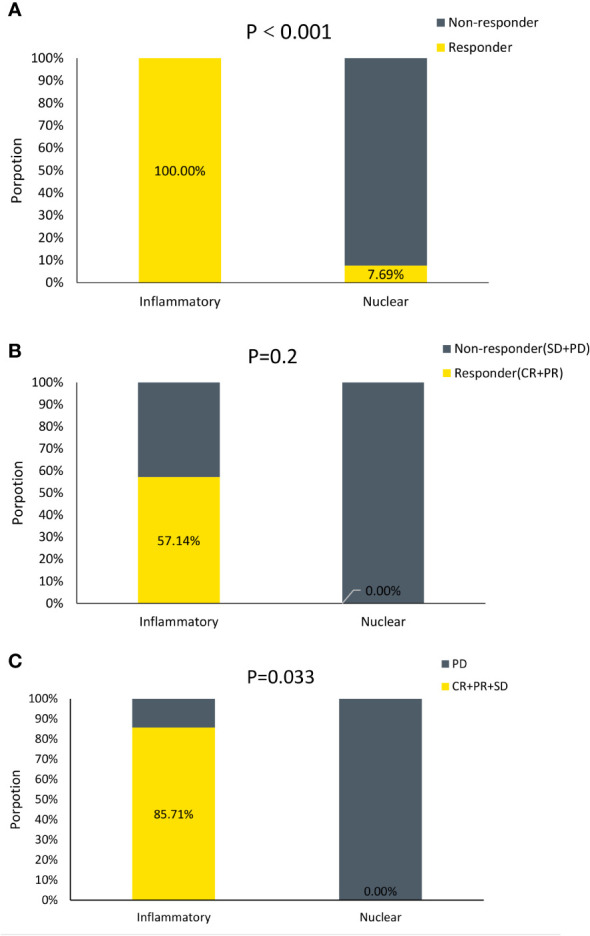
DAMPs-associated clusters can predict immunotherapy outcome. **(A)** Proportion of Responder and Non-responder to ICIs in mice. **(B)** Proportion of Responder and Non−responder to ICIs in MPM patients. **(C)** Proportion of Disease Control (CR+PR+SD) and Disease Progression (PD) to ICIs in MPM patients.

Then the efficacy of ICIs treatment was validated in a murine cohort and a MPM patient cohort treated with ICIs. The inflammatory DAMPs subtype showed a greater response rate than the nuclear DAMPs subtype in the validation cohort of 48 MPM mice receiving PD-L1 inhibitor combined with CTLA-4 inhibitor (100% vs. 7.7%, *P*<0.001; **Figure 9A**). The response rate of the two subtypes from the validation cohort of 10 MPM patients receiving PD-1 inhibitor as a single agent suggested a similar trend to the results of the murine cohort but failed to achieve statistical significance (57.1% vs. 0%, *P*=0.2; **Figure 9B**), while the disease control rate of the two subtypes was deemed to be statistically significant (85.7% vs. 0%, *P*=0.033; **Figure 9C**). These findings indicated that the developed DAMPs-associated clustering is capable of forecasting the efficacy of ICBs in MPM.

## 4 Discussion

Our study categorized MPM patients into two subgroups based on DAMPs and PRRs. In the nuclear DAMPs subtype, nuclear-associated HSP90AA1, HSPA4, CALR and HMGN1 were strongly expressed in the nuclear DAMPs subtype, whereas PRRs modulating activities of inflammasome or immune cells, such as TLRs, AIM2 and NLRP3, were primarily expressed in the inflammatory DAMPs subtype.

HMGN1(also known as alarmin), as a member of the high-mobility group protein family, is activated in undifferentiated cells which proliferate continuously. Extracellular HMGN1 functions as an innate danger-associated inflammatory mediator directly inducing the generation of cytokine and DC maturation. Upon translocation to the cytoplasm, it binds to PRRs to initiate proinflammatory signaling. Increasing expression of HMGN1 might provoke chronic inflammation contributing to carcinogenesis and indicating a poorer prognosis (55). The intracellular functions of CALR as a crucial regulator of Ca2+ homeostasis and the integrin-dependent signaling is probably required for tumor progression (56) which therefore implies that CALR expression is robustly related to prompt tumor progression and poor prognosis (57, 58) in the nuclear DAMPs subtype. HSPs in cells are essential in protein folding. The enriched Notch signaling positively regulate the activity of the mTOR pathway (59), and mTOR complex 1 (mTORC1) activates MYC-induced protein synthesis (60). Extreme endoplasmic reticulum (ER) stress and UPR are triggered by the uncontrolled buildup of misfolded proteins in the ER, which induce biological effects *via* upregulation of molecular chaperones such as HSP (e.g., HSP90AA1 and HSPA4). Elevated expression of HSPA4 and HSP90AA1 is related with poor clinical outcomes in cancer patients (61).

However, DAMPs alone are insufficient to elicit an ICD, and a corresponding receptor is required to generate biological effects. Toll-like receptors (TLRs), an evolutionarily conserved transmembrane protein expressed in epithelial cells and immune cells, serve as an important receptor to identify the DAMPs. TLRs bind with ligands such as HMGN1, CALR, HSPA4 and HSP90 to generate the affiliated biological effects *via* activation of MyD88-dependent and independent pathways. AIM2 and NLRP3 are sensors of DNA released from necrotic cells or increased Ca2+ release that initiate the inflammasome assembly (62, 63). Interleukin (IL)-1β and IL-18 are the inflammasome effector cytokines released as a result of signaling pathways that are regulated by inflammasomes. These cytokines assure an optimum inflammatory immune response against cancer cells (64). Therefore, MPM belonging to nuclear DAMPs subtype with higher expression of nuclear-associated DAMPs genes and lower corresponding PRRs expression are more likely to exhibit more aggressive biological behaviors. Nuclear-associated DAMPs are needed to regulate the progression of nuclear DAMPs subtype of MPM, which in consequence leads to poor outcomes. Under the circumstances of PRRs deficiency, the downstream signaling pathway cannot be activated, even with the presence or overexpression of DAMPs, causing effector cells suppressed and adapted immunity muted, which probably accounts for the suppressed immune microenvironment of the nuclear DAMPs subtype and primary resistance to immunotherapy, consistent with the predicted results of our survival analysis and exploration of immune status and ICIs efficacy.

The underlying biological pathways were then explored with functional analyses. Based on the DEGs, GSEA and GSVA identified inflammatory DAMPs subtype considerably enriched in the pro-inflammatory pathways that enhance adaptive immune responses. Apart from the toll-like receptor signaling pathway in line with higher expression of TLRs, TNFR binding and regulation of type I interferon-mediated signaling generates the antiproliferative effects of type I IFNs and TNFs (65) *via* activating the proinflammatory NF-κb pathway (66). Cytokine/chemokines pathways (such as IL-2 family, IL-12, IFN-γ, TNF superfamily, chemokine signaling, and IL6-JAK-STAT signaling) and effector immune cell pathways (such as NKT, DC, and T cell activation) were also significantly overexpressed. Upregulated IFN-γ signaling amplifies the antitumor response by mediating induced effects of IL-12 (67) and produces chemokines that attract immune effector cells, effectively changing the TME (68). The release of IFN-γ also leads to increasing antigen presentation of cancer and noncancer cells. Inflammatory chemokines induce recruitment of monocytes and help to support and regulate activated T cells (69). IL-2 mainly produced by CD4+ T cells activates effector T cells and innate lymphoid cells (ILCs) (70). The cytokines aforementioned regulate pro-inflammatory immunity by linking intrinsic and adaptive immune responses. Furthermore, ATM pathway, integrin A4B1 pathway and FGF pathway are enriched in this subtype. ATM (Ataxia-telangiectasia mutated proteins) is a key regulator of the DNA damage response (DDR) and contributes to cell cycle checkpoint maintenance, DNA damage repair and telomere maintenance in DNA double-strand breaks (DSB). The ATM pathway also regulates the suppression of anti-tumor immune cancer-associated fibroblasts (CAFs) differentiation (71). Advanced desmoplasia and stromal changes caused by CAFs have been identified as substantial factors in the progression of MPM (72). Accordingly, inhibition of ATM has the potential to overcome immune resistance in combination with ICIs in a minor subset of MPM patients of inflammatory DAMPs subtype. Fibroblast growth factors (FGFs) and integrin-α4β1 pathways are involved in oncogenic behaviors such as metastasis, angiogenesis, and activation of CAFs (73–75). Thereby, inhibitors targeting FGFs or integrin A4B1, or administrating anti-angiogenic agents may introduce promising directions for the management of this subtype. For the nuclear DAMPs subtype, GSEA revealed that expression of cancer hallmarks of MYC targets, NOTCH signaling, TGF-β signaling, unfolded protein response (UPR), MTORC1 signaling, and IL-8 production was higher in the nuclear DAMPs subtype. MM cells are dependent on Notch signaling, leading to activation of the phosphatidylinositol 3-kinase (PI3K)/Akt/mammalian target of rapamycin (mTOR) signaling pathway (76). NOTCH signaling facilitates immune escape by up-regulating PD-L1 and is associated with the expansion of exhausted CD8+ T cells (76). Expression profiles of malignant mesotheliomas revealed that 46% displayed altered expression of RPTOR (mTORC1 component) that activates the mechanistic target of rapamycin complex 1 (mTORC1) to enhance MM cell growth (77) suggesting the worse outcome of nuclear DAMPs subtype. On the other hand, studies have demonstrated the potential role of IL-8 as a driver of resistance to ICIs and that IL-8 has an essential role in reinforcing the immunosuppressive microenvironment and triggering EMT by determining the types and quantity of myeloid cells infiltrating tumors (78, 79). TGF-β is correlated with suppressing T cell proliferation and activation, impairing DC and NK cell function, encouraging Treg cell differentiation, and boosting CAF activities, ultimately gives rise to resistance to the immunotherapy (80) in the nuclear DAMPs subtype. Thus, targeting the aforementioned pathways is a plausible way to modify the suppressive immune microenvironment and provides new therapeutic options for this subtype.

The components in the tumor immune microenvironment (TiME) provide clues to predict MPM patient outcomes and ICIs responses (81). Activated memory CD4+ T cells, M1-like macrophages, and activated DCs were more prevalent in the inflammatory DAMPs subtype according to the CIBERSORT, while the ESTIMATE revealed that a significantly higher immune score, which have positive relations with better outcomes and more benefits from ICIs. Comparatively, M2-like macrophages of the nuclear DAMPs subtype were massively recruited, which have recently been discovered to participate in promoting resistance to ICIs therapy and predicting a poor outcome (82, 83). Furthermore, the improved OS and response rate to ICIs in the inflammatory DAMPs subtype may be associated with the dramatically elevated expression of CD8A and PD-1. These findings support the previous report that MPM patients with increased expression of CD8A and PD-1 enjoy a favorable prognosis and potentially benefit from ICIs in preceding clinical trials (84) (85). The expression of MHC class I and class II protein, particularly human leukocyte antigen class I (HLA-I) alleles, and proinflammatory chemokines and immunomodulators, especially CXCL10 and CXCR3, is more robust in the inflammatory DAMPs subtypes than the other. MHC expression on tumor cells from treatment-naive patients positively correlates with the clinical outcome and response to anti-CTLA-4, anti-PD-1, or their combination by recognizing tumor-specific antigens (86). In respect of CXCL10 and CXCR3 as downstream adjuvant effectors of type I IFNs, their signaling boosts the efficacy of immunotherapy by increasing immune infiltration of cytotoxic lymphocytes (CTLs), natural killer cells (NKs) and DCs (87). Therefore, upregulated CXCL10 and CXCR3 are positively linked with the efficacy of immunotherapy (88, 89). The above results laid further foundations for our reasonably predicting the better prognosis and response rate of ICIs treatment in the inflammatory DAMPs subtype than the other.

In the TCGA cohort, mutations of *TTN* were found more frequently in the inflammatory DAMPs subtypes and mutations of *TP53* in the nuclear DAMPs subtypes were clinical outcome and efficacy on the trend. Previous studies showed that patients with mutated *TTN* are associated with longer progression-free survival or overall survival (90). TTN expression was favorably associated with the infiltration levels of effector T cells owning an inflammatory TiME and TMB in numerous tumor types, and therefore is linked with susceptibility to immune checkpoint inhibitors (91–93). We can infer that *TTN* may have a connection with clinical outcomes and efficacy of immunotherapy as our model projected (94). As for TP53 mutation implying a more malignant nature, the nuclear DAMPs subtype is indicated to require more intense management (95).

Copy number deletion in MPM is a characteristic genetic alteration that may result from altered methylation caused by external factors such as asbestos. Copy number deletions of cancer suppressor genes (including CDKN2A/B, FOCAD, NF2, etc.) are always accompanied by adjacent functional genes (including MTAP, MLLT3, etc.) deletion that synergistically contributes to oncogenesis. Studies have shown that copy number deletion is associated with loss of tumor neoantigens and reduced gene expression of immune-related pathways (96), which predicts dismal immune efficacy in this subtype as our subtyping model does (97). Copy number loss of Cyclin-dependent kinase (CDK) inhibitor 2A (CDKN2A) occurs at a significantly higher frequency in the nuclear DAMPs subtype. CDKN2A copy number loss suggests dismal outcomes and predicts immunotherapy resistance (98). CDKN2A encodes p16INK4a which regulates cell-cycle by inhibition of CDK4/6. Emerging clinical data demonstrates selective CDK4/6 inhibitors widely used in clinical practice contribute to PD-L1 up-regulation and immune surveillance enhancement. Hence the combination of ICIs and CDK4/6 inhibitors is a worthwhile strategy for improving outcomes in the immunotherapy-tolerant nuclear DAMPs subtype of MPM. Fifty-seven percent of patients with CDKN2A copy number loss had methylthioadenosine phosphorylase (MTAP) co-deletion in the TCGA validation cohort, since both genes reside in the same cluster of the 9p21 region (99). Co-deletion occurs more frequently indicating poorer prognosis in nuclear DAMPs subtype than the other (72% vs 48%, P=0.032). Studies show that methionine adenosyltransferase 2A (MAT2A) inhibitors induce synthetic lethality of MTAP-deleted cancer, especially in combination with taxanes and gemcitabine (100), which demonstrate a potential to treat MPM of the nuclear DAMPs subtype. Accordingly, distinctive copy number deletion also offers novel approaches to the management of the immune-resistant subtype of MPM.

The antigenicity and adjuvanticity determine the immunogenicity of cell death. Despite MPM is characterized by genetic alterations in tumor suppressor genes (101, 102) suggesting a lack of tumor-associated antigens (TAAs) and a low level of tumor mutation burden (TMB) (103), studies showed that the lowest antigenicity associated with tumorigenesis might be minor but is sufficient to support immunogenicity (12). In this regard, the adjuvanticity of DAMPs and their PRRs in immunogenic cell death may constitute a promising target for activating the immune response, since it is in a superior position to preserve homeostasis of immune microenvironment. Counteracting the inhibition of DAMPs and PRRs (e.g., TLRs stimulators) may suppress tumor growth as well as balance the production of cytokines within the TiME, and further, suppress the immunosuppressive cells while activating the immunostimulatory or effector cells (12), and thus the efficacy of immunotherapy can be enhanced by intervening with the immunomodulatory effects of DAMPs and its downstream signaling. According to the perspectives above, pathways and genomic alterations more peculiar in the nuclear DAMPs subtype of MPM which is primarily resistant to ICIs can be manipulated to modulate the immune status to some extent.

However, there are drawbacks to our study. Firstly, a few genes in the established gene list were excluded due to limitations of expression sequencing by microarray, yet the modified gene set was representative of the concerned genes in the process of ICD. Secondly, there is insufficient sequencing data on patients treated with ICIs since mesothelioma is a rare tumor. Models constructed of mice of the same strain (BALB/c) with identical genetic backgrounds inoculated subcutaneously with the same cell lines (AB1-HA cells) and then treated with anti-CTLA-4 and anti-PD-L1 were selected for additional validation. Moreover, the ORR of PD-1 inhibitors applied to MPM patients of two separate subtypes in the validation cohort did not reach statistical significance, although there was a trend that implied patients belonging to the inflammatory subtype benefit more from ICIs and the DCR, probably on account of small sample size or limited efficacy of ICIs as a single agent in MPM. Additional research is expected to confirm the validity and clinical practicability in larger cohorts or elaborately designed clinical trials.

## 5 Conclusion

In conclusion, we identified two molecular subtypes *via* K-means analysis based on the expression of ICD-associated DAMPs and their corresponding receptors in MPM. Characteristic signaling pathways and different immune statuses in these two subtypes result in disparate prognoses and efficacy of immune checkpoint inhibitors.

Our research offers a novel method to predict the prognoses and identify the MPM patients with a potential to benefit from ICIs and provides a new perspective to enhance the efficacy of immunotherapy for MPM patients with primary resistance to ICIs. Our work has made a step forward in the process of development of precision therapy in MPM.

## Data availability statement

The datasets presented in this study can be found in online repositories. The names of the repository/repositories and accession number(s) can be found within the article/supplementary materials.

## Author contributions

ZL: conceptualization, methodology, software, data curation, investigation, validation, visualization, writing- original draft preparation, writing- reviewing and editing. HB: supervision, funding acquisition. RW: writing- reviewing and editing. JW: conceptualization, supervision, writing- reviewing and editing, funding acquisition. All authors contributed to the article and approved the submitted version.
